# Biocompatible Casein Electrolyte-Based Electric-Double-Layer for Artificial Synaptic Transistors

**DOI:** 10.3390/nano12152596

**Published:** 2022-07-28

**Authors:** Hwi-Su Kim, Hamin Park, Won-Ju Cho

**Affiliations:** 1 Department of Electronic Materials Engineering, Kwangwoon University, Gwangun-ro 20, Nowon-gu, Seoul 01897, Korea; hwisu0811@naver.com; 2 Department of Electronic Engineering, Kwangwoon University, Gwangun-ro 20, Nowon-gu, Seoul 01897, Korea; parkhamin@kw.ac.kr

**Keywords:** synaptic transistor, casein, biocompatible, organic material, electric-double-layer, artificial synapses, neuromorphic computing

## Abstract

In this study, we proposed a synaptic transistor using an emerging biocompatible organic material, namely, the casein electrolyte as an electric-double-layer (EDL) in the transistor. The frequency-dependent capacitance of the indium-tin-oxide (ITO)/casein electrolyte-based EDL/ITO capacitor was assessed. As a result, the casein electrolyte was identified to exhibit a large capacitance of ~1.74 μF/cm^2^ at 10 Hz and operate as an EDL owing to the internal proton charge. Subsequently, the implementation of synaptic functions was verified by fabricating the synaptic transistors using biocompatible casein electrolyte-based EDL. The excitatory post-synaptic current, paired-pulse facilitation, and signal-filtering functions of the transistors demonstrated significant synaptic behavior. Additionally, the spike-timing-dependent plasticity was emulated by applying the pre- and post-synaptic spikes to the gate and drain, respectively. Furthermore, the potentiation and depression characteristics modulating the synaptic weight operated stably in repeated cycle tests. Finally, the learning simulation was conducted using the Modified National Institute of Standards and Technology datasets to verify the neuromorphic computing capability; the results indicate a high recognition rate of 90%. Therefore, our results indicate that the casein electrolyte is a promising new EDL material that implements artificial synapses for building environmental and biologically friendly neuromorphic systems.

## 1. Introduction

Recently, with increasing unstructured data including videos and images, the importance of information processing ability has risen in big data and artificial intelligence fields [1,2,3]. However, traditional computing systems, such as the von Neumann architecture, faced high power consumption and low-speed processing challenges due to a bottleneck caused by the physical separation of memory and computation parts when processing large amounts of data [4,5]. Accordingly, a computing system that is more efficient than conventional computing systems in terms of power and processing speed is urgently required. The human brain can simultaneously perform complex computations and memories at low power (~20 W) with a highly parallel and fault-tolerant neural network involving approximately 10^11^ and 10^15^ neurons and synapses, respectively [6,7]. Therefore, studies on neuromorphic systems, which are brain-like computing structures, are receiving a lot of attention [8,9]. Biological synapses, nanogaps connecting two neurons in the brain nervous system, are a core part of signal transmission and functional connections. Synaptic plasticity is a major biological function that contributes to multidimensional information learning, memory, and processing in the brain, and it is caused by the changes in synaptic weight, which can be modulated by the flow of ions through the synapse [10,11]. Therefore, electronic devices that can mimic biological synaptic behavior are important for implementing neuromorphic systems [12,13]. In the past few years, various types of two- and three-terminal artificial synaptic devices have been proposed to implement synaptic functions with low energy consumption, including memristors, phase change memory, ferroelectric devices, and field-effect transistors (FETs) [14,15,16,17]. Particularly, the electrolyte gate transistor successfully emulates synaptic functions by the electric-double-layer (EDL) formed at the gate electrode/electrolyte and electrolyte/channel layer interfaces; this is also referred to as an EDL transistor. The internal migration of ions within electrolytes in response to the bias on gate electrodes contributes to the modulation of channel conductance, which is considered the synaptic weight. The EDL transistor has a large capacitance (>1 μF/cm^2^) due to the EDL effect based on mobile ions, resulting in low power consumption, as a large amount of charge is induced in a channel with a low driving voltage [18,19]. Meanwhile, the increased interest in eco-friendly and bio-non-hazardous electronic devices has resulted in the active research of bio-materials these days. Consequently, several EDL synaptic transistors developed using various natural organic materials, such as chitosan, cellulose, starch, and albumin, as electrolytes have been reported [20,21,22,23]. These organic materials are suitable for biocompatible synaptic transistor applications as they are abundant, biodegradable, non-toxic, and capable of low-cost solution processing [24].

Previously, we demonstrated the neuromorphic behavior of synaptic transistors using commercial bovine milk as EDL [25]. In this study, we propose an EDL synaptic transistor using casein electrolyte as a biocompatible organic material with a high potential for artificial synapses. Casein, a protein component that accounts for a large portion of milk, is a naturally sufficient biocompatible organic material. It is widely used in diverse fields, such as food, industry, and medical care [26,27,28,29]. We verified the EDL effect by protons in casein electrolytes, which play an important role in the synaptic operation, by evaluating frequency-dependent capacitance. Subsequently, the synaptic transistors were fabricated using an indium-tin-oxide (ITO) bottom gate, casein electrolyte-based EDL gate insulator, and indium-gallium-zinc oxide (IGZO) channels as the pre-synapse, neurotransmitter, and post-synapse, respectively. The transfer and output characteristics of the fabricated synaptic transistor were evaluated. Additionally, the migration of mobile protons inside the casein electrolyte was confirmed through the change in hysteresis according to the gate voltage range. Furthermore, major properties related to the synaptic plasticity, such as single-spike excitatory post-synapse current (EPSC), paired-pulse facilitation (PPF), frequency-dependent EPSC, potentiation/depression, and spike-timing-dependent plasticity (STDP), were successfully demonstrated. Finally, a handwritten Modified National Institute of Standards and Technology (MNIST) pattern recognition was simulated based on a three-layer artificial neural network (ANN) to confirm the potential application of the proposed transistor in neuromorphic systems.

## 2. Materials and Methods

### 2.1. Materials

Corning 7509 glass substrate (Corning Inc., Corning, NY, USA), casein powder derived from bovine milk (technical grade, Sigma-Aldrich, Seoul, Korea), an acetic acid solution (purity > 99%, Sigma-Aldrich), IGZO sputter target (In_2_O_3_:Ga_2_O_3_:ZnO = 4:2:4.1 mol%, THIFINE Co., Ltd., Incheon, Korea), and ITO sputter target (In_2_O_3_:SnO_2_ = 9:1 mol%, THIFINE Co., Ltd., Korea) were the materials used in this study. 

### 2.2. Preparation of Casein Solution

**Figure 1:**
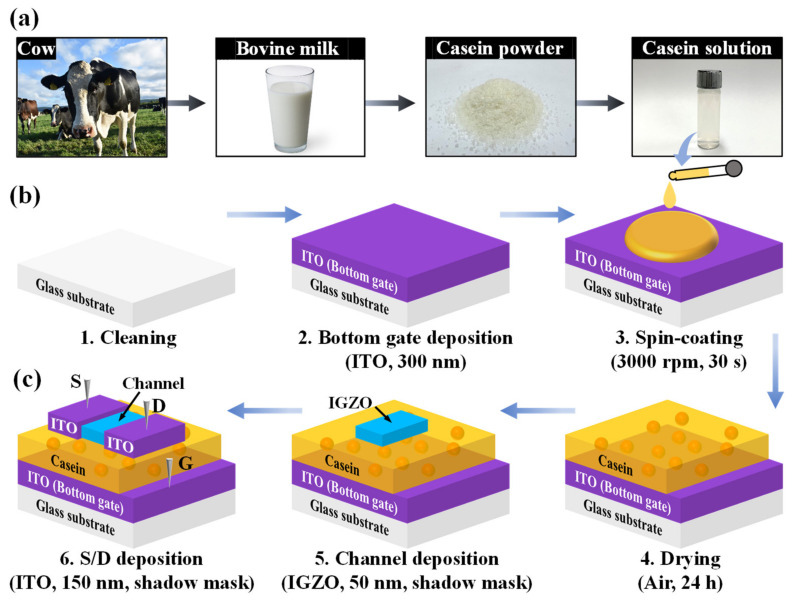
(**a**) Schematic of casein solution preparation. (**b**) Fabrication details and (**c**) schematics of casein electrolyte-based electric-double-layer (EDL) synaptic transistors on glass substrates.

A biocompatible casein solution was prepared by dissolving a mixture of casein powder and acetic acid solution. Herein, 3 wt% casein powder derived from bovine milk (technical grade, Sigma-Aldrich) was added to a 3 wt% acetic acid solution with purity > 99% (Sigma-Aldrich) and diluted with deionized water. The solution was then constantly mixed and completely dissolved using a magnetic stirrer with 800 rpm for 6 h at 130 °C. Finally, the solution was filtered through a polytetrafluoroethylene syringe filter with a 5 μm pore size (Whatman International Ltd., Maidstone, UK) to eliminate impurities. Figure 1a depicts the prepared casein solution.

### 2.3. Fabrication of Casein Electrolyte-Based EDL Synaptic Transistors

Figure 1b illustrates the fabrication of the casein electrolyte-based EDL synaptic transistors. After cleaning the surfaces of the transparent Corning 7509 glass substrates with dimensions of 1 × 1 cm^2^ as a starting material, a bottom gate electrode was formed by depositing a 300 nm-thick ITO film using radio frequency (RF) magnetron sputtering. The ITO sputtering process was performed at a working pressure of 3 mTorr, an RF power of 100 W, and an Ar flow rate of 20 sccm. The casein solution, which plays a key role as EDL in synaptic transistors, was then spin-coated at 3000 rpm for 30 s and dried in air conditioning at room temperature (25 °C) for 24 h. Subsequently, a 50 nm-thick IGZO channel layer was deposited using a shadow mask via RF magnetron sputtering. The working pressure, RF power, and an Ar flow rate of the IGZO sputtering process were 6 mTorr, 100 W, and 30 sccm, respectively. Finally, a 150 nm-thick ITO film was deposited for source/drain (S/D) electrodes using the shadow mask via RF magnetron sputtering. In fabricated synaptic transistors, the channel and S/D width (W) × length (L) were 1000 μm × 80 μm and 1000 μm × 200 μm, respectively. Figure 1c shows the schematic of the fabricated casein electrolyte-based EDL synaptic transistor.

### 2.4. Characterizations

The frequency-dependent capacitance of the ITO/casein electrolyte-based EDL/ITO capacitors was characterized using an Agilent 4284A Precision LCR meter (Agilent Technologies, Santa Clara, CA, USA). The electrical characteristic and synaptic functions of the fabricated casein electrolyte-based EDL transistors were analyzed using an Agilent 4156B Precision Semiconductor Parameter Analyzer (Agilent Technologies). To verify synaptic functions, electrical pre- and post-synaptic spikes were applied using an Agilent 8110A Pulse Generator (Agilent Technologies). All electrical evaluations were performed on a probe station in a dark box to prevent the effect of external light and electrical noise.

## 3. Results

### 3.1. Verification of Casein Electrolyte Properties for EDL

**Figure 2:**
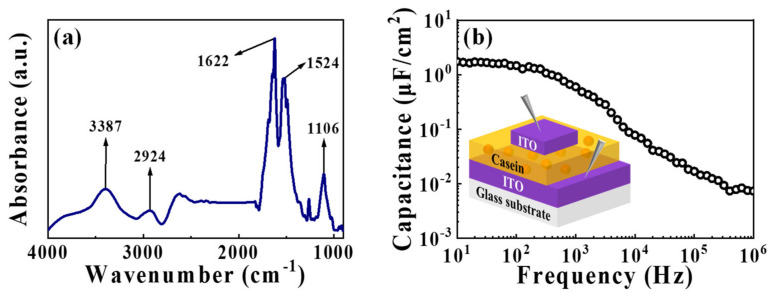
(**a**) Fourier transform infrared spectroscopy (FT-IR) spectrum of the casein electrolyte film. (**b**) Capacitance-frequency (C-*f*) curve of the metal-insulator-metal (MIM) structured capacitors (indium-tin-oxide (ITO)/casein electrolyte-based EDL/ITO).

The various properties of casein electrolyte must be verified before applying it to synaptic transistors. Fourier transform infrared spectroscopy (FT-IR) was performed to identify the composition of the casein electrolyte membrane that was sufficiently dried in an air environment. Figure 2a indicates the FT-IR spectrum of the casein electrolyte film in the range of 4000 to 900 cm^−1^, wherein the broad peaks at approximately 3387 and 2924 cm^−1^ are caused by the O-H and C-H stretching, respectively. The region between 1700 and 1600 cm^−1^ is associated with the amide I region, and the peak near 1622 cm^−1^ is attributed to the C=O stretching. Additionally, the area from 1600 to 1500 cm^−1^ is related to the amide II region, and the corresponding peak near 1524 cm^−1^ is attributed to the N-H bending and C-N stretching. Moreover, the peak at around 1106 cm^−1^ is due to the C-O stretching [30,31,32,33,34]. In general, the peaks associated with the amide and -OH group are primarily observed in the FT-IR spectrum of the casein electrolyte film. Amide groups are a major component of proteins and are detected as casein peaks that are included in the preparation of electrolytes. Casein accounts for approximately 80% of milk protein and is reported to be rich in mobile protons [25]. Furthermore, the presence of -OH groups contributes to the facilitation of proton conduction [22,23]. Therefore, the mobile protons can cause ion conduction in the casein electrolyte, resulting in the formation of EDLs at the casein electrolyte interface.

The EDL with a large capacitance exhibits a strong coupling effect of the gate to the channel. It aids in constructing energy-efficient artificial synapses by adjusting the ions and conductivities even at small voltages [35,36]. Therefore, to evaluate the EDL effect of the casein electrolyte, we measured the frequency-dependent capacitance characteristics of casein electrolytic capacitors with the metal-insulator-metal (ITO/casein electrolyte-based EDL/ITO) configuration. Figure 2b depicts the capacitance measured over a wide frequency range of 10 Hz to 1 MHz. The capacitance increased as the frequency decreased from 1 MHz, resulting in a large capacitance of ~1.74 μF/cm^2^ at 10 Hz. This change in capacitance is related to the frequency-dependent response of internal mobile protons. At high frequencies, the insufficient response time for protons to reach the interface results in low capacitance. Conversely, the sufficient response time at low frequencies facilitates the accumulation of protons at both interfaces. Consequently, an EDL that can be considered a nanometer-thick capacitor is formed, generating a high parallel plate capacitance [18,37]. Therefore, the frequency-dependent capacitance characteristics verify the EDL effect of casein electrolyte and suggest the possibility of application to EDL-based synaptic devices.

### 3.2. Electrical Characteristics of Casein Electrolyte-Based EDL Synaptic Transistors

**Figure 3:**
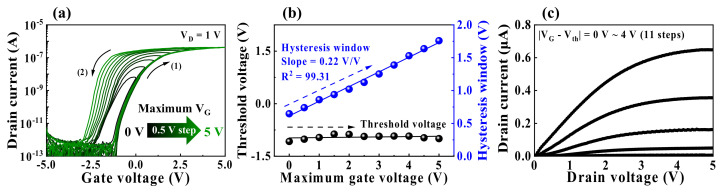
(**a**) Double-sweep transfer curves versus maximum gate voltage (0 to 5 V in steps of 0.5 V) at a constant V_D_ = 1 V. (**b**) Hysteresis window and threshold voltage with each double-sweep transfer curve. (**c**) Output curves as a function of gate voltage–threshold voltage (V_G_–V_th_ = 0−4 V in 0.4 V steps).

Figure 3a shows the double-sweep transfer curves (I_D_–V_G_) of the fabricated casein EDL synaptic transistors, where the maximum gate voltage (V_G_) was increased from 0 to 5 V in steps of 0.5 V at a constant drain voltage (V_D_) of 1 V. The transfer curve exhibits counter-clockwise hysteresis as the V_G_ switches to the backward sweep (2) after the forward sweep (1). Additionally, the larger the maximum V_G_, the larger the hysteresis window. This hysteresis characteristic is attributed to the slow polarization caused by the migration of mobile protons in the casein electrolyte-based EDL. The larger the maximum V_G_ in the forward sweep, the higher the number of mobile protons that accumulate at the interface of the IGZO channel/casein electrolyte-based EDL, which in turn induces more charge carriers in the channel layer. Subsequently, the protons gradually diffuse back in the opposite direction in the backward sweep, increasing the counter-clockwise hysteresis window. Figure 3b depicts the hysteresis window and threshold voltage (V_th_) extracted from the double-sweep transfer curve corresponding to the maximum V_G_. As a result of increasing the maximum V_G_ from 0 to 5 V, V_th_ remained nearly constant at −0.95 V. Meanwhile, the hysteresis window increased from 0.64 to 1.76 V with a slope of 0.22 *v*/*v* and linearity (R^2^) of 99.31. Figure 3c shows the output curve (I_D_–V_D_) measured in steps of 0.4 V as a function of V_G_–V_th_ from 0 to 4 V. The drain current (I_D_) increases linearly with the increase in V_D_; I_D_ gradually saturates, exhibiting typical pinch-off characteristics.

### 3.3. Synaptic Properties of Casein Electrolyte-Based EDL Synaptic Transistors

**Figure 4:**
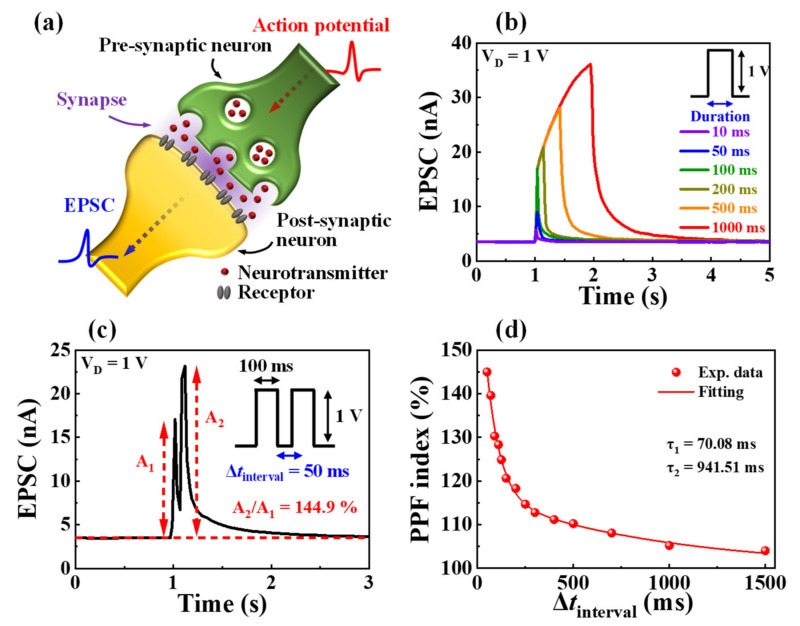
(**a**) Schematic of a biological synapse. (**b**) Excitatory post-synaptic current (EPSC) generated by a single pre-synaptic spike fixed amplitude (1 V) over various durations (10 to 1000 ms). (**c**) Paired pulse facilitated EPSC by a paired pre-synaptic spike (1 V, 100 ms) at Δ*t*_interval_ = 50 ms. (**d**) Paired pulse facilitation (PPF) index (A_2_/A_1_) plotted against various spike time intervals (Δ*t*_interval_) from 50 to 1500 ms.

Figure 4a is a schematic illustration of a biological synapse in the brain. Synapses are nano clefts between pre- and post-synaptic neurons that are crucial in signal transduction [38]. The pre-synaptic neuron input is transmitted to post-synaptic neurons through the diffusion of intrasynaptic neurotransmitters, generating transient currents, EPSCs. EPSC is the basic representation of synaptic weight, which can be modulated by adjusting the ion flux [39]. To implement synaptic transistors, it is essential to imitate the mechanisms and structures of biological synapses. The fabricated casein electrolyte-based EDL synaptic transistor considered the ITO bottom gate, IGZO channel, and mobile protons in the casein-based EDL as the pre-synapse, post-synapse, and neurotransmitter, respectively. Moreover, the channel current generated by the electrical stimulation of the pre-synapse gate is regarded as EPSC, and the S/D ITO electrode is used to read the EPSC. Figure 4b shows the single-spike EPSC characteristic curves with a pre-synaptic spike amplitude of 1 V for various durations from 10 to 1000 ms with a constant V_D_ = 1 V. The longer the pre-synaptic spike duration, the higher the maximum EPSC value at the end of the spike stimulus, and the longer the EPSC retention time after the spike stimulus. This is because the channel conductivity and concentration gradient increase as more mobile protons of the casein electrolyte migrate to the casein electrolyte/channel interface during longer spike durations. These results indicate that the modulation of synaptic plasticity can be implemented by changes in the channel conductance of the proposed casein electrolyte-based EDL synaptic transistors.

In the nervous system, PPF represents short-term synaptic plasticity and is important for decoding biological temporal information, including auditory or visual data [40]. PPF is a function of the synaptic spike time interval (Δ*t*_interval_), where the post-synaptic potential or current induced by the second pre-synaptic spike is amplified when the second spike closely follows the first spike [20]. Figure 4c depicts the EPSCs triggered by two identical pre-synaptic spikes with an amplitude of 1 V and a duration of 100 ms applied at a spike time interval of 50 ms; here, the second EPSC was increased when compared to the first. When the spike time interval is shorter than the charge carrier relaxation time, the incompletely relaxed protons induced by the first spike contribute to the protons induced by the second spike, increasing the channel current [41]. The PPF index can be calculated as the ratio of the first EPSC (A_1_) and second EPSC (A_2_), as shown in Figure 4d. The PPF index was given a high value of ~144.98% for short spike time intervals of 50 ms; however, the value decreased to ~104.02% for long spike time intervals of 1500 ms. The obtained PPF index was fitted using the following double exponential decay relationship indicated [42]:

(1) $$\mathrm{PPF}\;\mathrm{index}=C_{1}\exp\left(-\Delta t_{\mathrm{interval}}/\tau_{1}\right)+C_{2}\exp\left(-\Delta t_{\mathrm{inverval}}/\tau_{2}\right)+1$$

where *C*_1_ and *C*_2_ denote the initial facilitation magnitudes, and *τ*_1_ and *τ*_2_ indicate the characteristic relaxation times. In our transistor, the values of *τ*_1_ and *τ*_2_ were 70.08 and 941.51 ms, respectively, which are almost identical to those of typical biological synapses [41,42].

**Figure 5:**
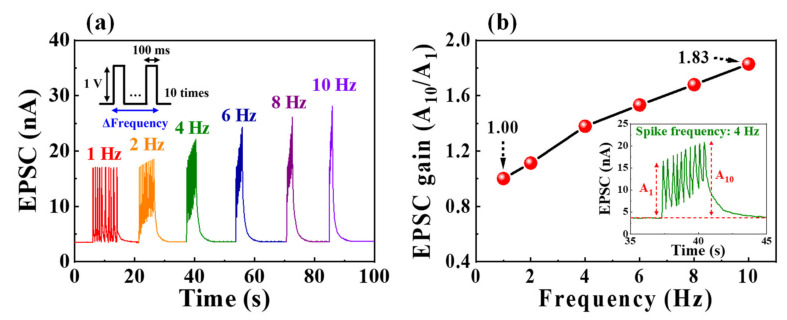
(**a**) EPSC responses generated by sequential pre-synaptic spikes (1 V, 100 ms) with various frequencies from 1 to 10 Hz. (**b**) Pre-synaptic spike-frequency-dependent EPSC gains (A_10_/A_1_). Inset denotes the EPSC response to 4 Hz.

Furthermore, short-term synaptic plasticity may enable the function of a dynamic filter for information transmission depending on the signal frequency. Short-term synaptic depression and facilitation contribute to low- and high-pass temporal filtering functions, respectively [36,43]. Figure 5a shows the EPSC responses to ten sequential pre-synaptic spikes (1 V amplitude, 100 ms duration) with different frequencies from 1 to 10 Hz. At a frequency of 1 Hz, the EPSC value triggered by each spike remained almost constant at ~17 nA, similar to that triggered by the first spike. Conversely, we observed that the maximum EPSC value increased with the increase in frequency. Figure 5b depicts the pre-synaptic spike-frequency-dependent EPSC gain calculated as the ratio of the first EPSC (A_1_) to the tenth EPSC (A_10_). When the frequency increased from 1 to 10 Hz, the EPSC gain increased from 1 to 1.83, indicating the short-term synaptic facilitation. Therefore, the proposed synaptic transistor successfully mimics the high-pass time filter function.

**Figure 6:**
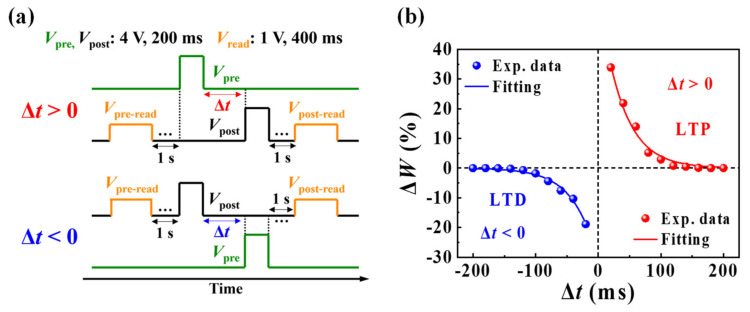
(**a**) Schematic of the spike sequence based on the relative timing difference between post- and pre-synaptic spikes (Δ*t*). (**b**) Synaptic weight change (Δ*W*) as a function of Δ*t*, which demonstrates the biological asymmetric Hebbian learning spike-timing-dependent plasticity (STDP) function.

In contrast to short-term plasticity, long-term plasticity exhibits long-term changes in synaptic weights and generates a memory storage model. The synaptic weight is strengthened by rapid and repetitive stimulation. The long-lasting increase in synaptic weight is referred to as the long-term potentiation (LTP), whereas the weakening of the synaptic weight over a long period is referred to as long-term depression (LTD) [44,45]. For our device, both LTP and LTD relative spike times were characterized by STDP. STDP is an improvement of Hebbian learning rules considering the temporal sequence of activities between pre- and post-synaptic neurons, which regulates the strength of connections between neurons via temporally correlated neural learning. Moreover, STDP is considered a major learning and memory mechanism in the brain [46,47,48]. Figure 6a illustrates the schematics of pre- and post-synaptic spikes with relative timing differences (Δ*t* = *t*_post_ − *t*_pre_) applied to STDP measurements. A pre-synaptic spike (*V*_pre_) and post-synaptic spike (*V*_post_) of 4 V were applied to the gate and drain, respectively, for 200 ms, and |Δ*t*| was set from 20 to 200 ms. The synaptic weight change (∆*W*) can be obtained using Equation (2) [48], as follows:

(2) $$\Delta W=\frac{I_{\mathrm{post}}-I_{\mathrm{pre}}}{I_{\mathrm{pre}}}\times 100\;\%$$

where *I*_post_ and *I*_pre_ denote the values of drain current measured using a pre-read pulse (*V*_pre-read_) and post-read pulse (*V*_post-read_), respectively, at 1 V for 400 ms on the drain (Figure 6a). Figure 6b shows the calculated ∆*W*, which is represented by dots. The result exhibits asymmetric Hebbian learning STDP characteristics, which is one of the biological STDP functions [49]. When |Δ*t*| decreased, synaptic weights were strengthened during the post-synaptic spike process after the pre-synaptic spike (Δ*t* > 0), resulting in LTP. By contrast, the synaptic weight was weakened during the pre-synaptic spike process after the post-synaptic spike (Δ*t* < 0), resulting in LTD. The calculated ∆*W* is well-fitted by the STDP learning function, as indicated by the solid lines in Figure 6b. The STDP learning function can be defined as [47]:

(3) $$\Delta W=\left\{\begin{array}{c} A^{+}e^{-\Delta t/\tau^{+}},\;\;\mathrm{if}\;\Delta t>0\\ -A^{-}e^{\Delta t/\tau^{-}},\;\;\mathrm{if}\;\Delta t<0 \end{array}\right.$$

where *τ*^±^ denotes the time constant that determines the range of Δ*t* over which synaptic strengthening and weakening occur; *A*^±^ indicates a scaling factor defining the maximum amount of synaptic weight modification that occurs when Δ*t* approaches zero. The obtained *A*^+^, *A*^−^, *τ*^+^, and *τ*^−^ are estimated to be 60.06%, 32.07%, 36.92 ms, and 37.50 ms, respectively. These results demonstrate that biological STDP behavior can be emulated in the proposed casein electrolyte-based EDL synaptic transistors.

**Figure 7:**
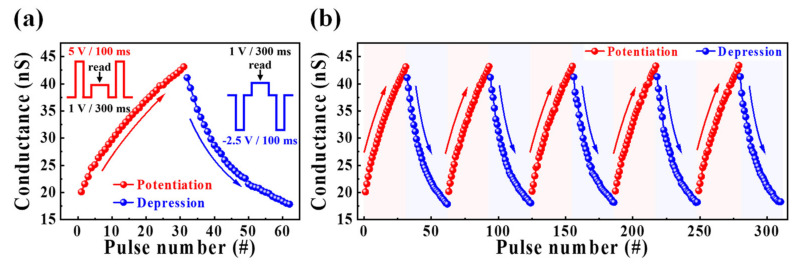
(**a**) Potentiation and depression characteristics of synaptic weights by applying pre-synapse pulses. (**b**) Endurance properties for five cycles of potentiation and depression.

Figure 7a shows the characteristics of the continuous channel conductance modulation for repetitive pre-synaptic stimuli; the insets illustrate a single cycle of potentiation/depression and read pulses. A single cycle of pre-synaptic stimulation consists of 30 potentiation pulses of 5 V for 100 ms and 30 depression pulses of −2.5 V for 100 ms applied to the bottom gate electrode. To measure the change in the channel conductance owing to the pre-synaptic stimulation, a read pulse of 1 V was applied to the drain for 300 ms. The conductance increased and decreased for the potentiation and depression pulses, modulating from 20.1 to 42.5 nS, and from 41.1 to 18 nS for the potentiation and depression behaviors, respectively. Figure 7b depicts the endurance characteristics for potentiation and depression over five cycles. It is found that the conductance modulation was maintained nearly constant. Therefore, the LTP and LTD properties induced by repetitive stimuli were validated in our synaptic transistors.

### 3.4. MNIST ANN Simulation

**Figure 8:**
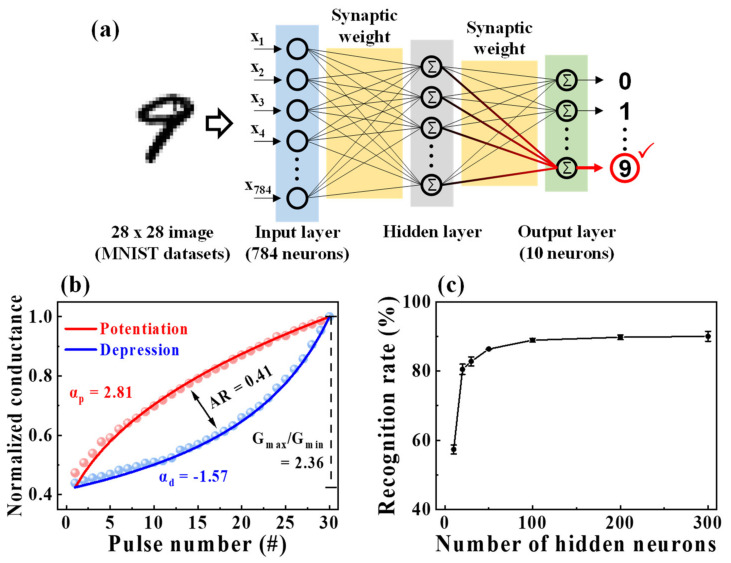
(**a**) Schematics of a three-layer fully connected artificial neural network (ANN) comprising the input, hidden, and output layers for handwritten Modified National Institute of Standards and Technology (MNIST) recognition. (**b**) Nonlinearity analysis on normalized potentiation and depression (G/G_max_). (**c**) Simulated recognition rate with different numbers of hidden neurons. The error bar indicates the standard deviation.

Finally, we designed a three-layer perceptron network model, which simulates the learning of MNIST handwritten digits to confirm the ability of neuromorphic computing in the proposed synaptic transistor. The developed ANN was composed of the input, hidden, and output layers, as shown in Figure 8a. The 784 input neurons in the input layer and 10 output neurons in the output layer corresponded to 28 × 28 pixels of binarized MNIST data and digits from 0 to 9, respectively. Each neuron was connected to another neuron in the subsequent layer through the synapse, and the strengths of the connection, as the synaptic weights, were related to the normalized potentiation and depression conductance of the casein electrolyte-based EDL synaptic transistor, as shown in Figure 8b. The normalized conductance was calculated by dividing each conductance by the maximum conductance (G/G_max_). In the accuracy of the learning and recognition simulation, the properties of potentiation and depression, such as the dynamic range (G_max_/G_min_), asymmetric ratio, and linearity, are important. The dynamic range (G_max_/G_min_) value, which represents the conductance modulation range, was 2.36. The symmetry of the increase in potentiation and the decrease in depression is indicated in asymmetric ratio as [50]:

(4) $$AR=\frac{max\left|G_{p}\left(n\right)-G_{d}\left(n\right)\right|}{G_{p\left(30\right)}-G_{d\left(30\right)}}\;\;for\;\;n=1\;to\;30$$

The closer the value to the ideal asymmetric ratio of zero, the higher the learning accuracy. The calculated asymmetric ratio was 0.41 for the proposed synaptic transistor. In addition, to confirm the linearity of conductance, the nonlinearity factor was extracted using the following equation [51]:

(5) $$G=\left\{\begin{array}{c} {\left(\left(G_{max}^{\alpha }-G_{min}^{\alpha }\right)\times w+G_{min}^{\alpha }\right)}^{1/\alpha },\;\;\mathrm{if}\;\alpha \neq 0\\ \;\;\;\;\;\;\;\;\;\;\;\;\;\;\;\;\;G_{min}\times {\left(\frac{G_{max}}{G_{min}}\right)}^{w}\;\;\;\;\;\;\;\;\;\;\;,\;\;\mathrm{if}\;\alpha =0 \end{array}\right.$$

where *G_max_* and *G_min_* donate the maximum and minimum conductance, respectively, and *w* is an internal variable (from 0 to 1). α represents a nonlinearity factor that controls the potentiation (α_p_) or depression (α_d_), and the ideal value of the nonlinearity factor is one. The obtained nonlinearity factors for potentiation and depression were 2.81 and −1.57, respectively, in the developed synaptic transistor. We designed the synaptic weight of the ANN utilizing the normalized conductance and extracted factors. Then, the ANN was trained using 60,000 MNIST training datasets, and the recognition test was conducted using 10,000 other test datasets. Figure 8c shows the recognition rate resulting in simulation when the number of neurons varies from 10 to 300 in the hidden layer. The recognition rate was 57% in 10 hidden neurons and increased as the neurons increased, achieving a sufficient recognition rate of 90%.

## 4. Conclusions

In summary, we used a biocompatible casein electrolyte as an EDL material for the fabrication of synaptic transistors. The EDL effect of casein electrolyte caused by abundant mobile protons was verified based on the large capacitance of ~1.74 μF/cm^2^ at 10 Hz and increase in capacitance with decreasing frequency through frequency-dependent capacitance characteristics. We evaluated the electrical characteristics and various functions of the biological synapses of casein electrolyte-based EDL synaptic transistors. In the double-sweep transfer curves, the counter-clockwise hysteresis window caused by the proton migration increased with the increasing maximum gate voltage. Furthermore, essential biological synaptic behaviors, including short- and long-term synaptic plasticity and high-pass time filtering capabilities, were successfully emulated through single-spike EPSC, PPF, frequency-dependent EPSC, STDP, and potentiation and depression characteristics. In addition, an excellent recognition rate of 90% was achieved for the handwritten MNIST learning simulation. Therefore, casein electrolyte, a promising EDL material implementing artificial synapses, is expected to provide potential applications for eco-friendly and biocompatible neuro-morphic systems.

## Data Availability

Not applicable.
